# Challenges, coping responses and supportive interventions for international and migrant students in academic nursing programs in major host countries: a scoping review with a gender lens

**DOI:** 10.1186/s12912-021-00678-0

**Published:** 2021-09-18

**Authors:** Lisa Merry, Bilkis Vissandjée, Kathryn Verville-Provencher

**Affiliations:** 1 Faculty of Nursing, University of Montreal, Montreal, Canada; 2 SHERPA Research Centre, The University Institute with Regards to Cultural Communities, CIUSSS West-Central Montreal, Montreal, Canada; 3 InterActions, Centre de recherche et de partage des savoirs, CIUSSS du Nord-de-l’Île-de-Montréal, Montreal, Canada; 4 Centre de recherche en santé publique (CReSP) du CIUSSS du Centre-Sud-de-l’Île-de-Montréal et l’Université de Montréal, Montreal, Canada

**Keywords:** International students, Nursing education, Foreign-born students, Migrant students, Gender, Gender identity, Coping responses, Supportive interventions, Scoping review, High-income countries

## Abstract

**Background:**

International and migrant students face specific challenges which may impact their mental health, well-being and academic outcomes, and these may be gendered experiences. The purpose of this scoping review was to map the literature on the challenges, coping responses and supportive interventions for international and migrant students in academic nursing programs in major host countries, with a gender lens.

**Methods:**

We searched 10 databases to identify literature reporting on the challenges, coping responses and/or supportive interventions for international and migrant nursing students in college or university programs in Canada, the United-States, Australia, New Zealand or a European country. We included peer-reviewed research (any design), discussion papers and literature reviews. English, French and Spanish publications were considered and no time restrictions were applied. Drawing from existing frameworks, we critically assessed each paper and extracted information with a gender lens.

**Results:**

One hundred fourteen publications were included. Overall the literature mostly focused on international students, and among migrants, migration history/status and length of time in country were not considered with regards to challenges, coping or interventions. Females and males, respectively, were included in 69 and 59% of studies with student participants, while those students who identify as other genders/sexual orientations were not named or identified in any of the research. Several papers suggest that foreign-born nursing students face challenges associated with different cultural roles, norms and expectations for men and women. Other challenges included perceived discrimination due to wearing a hijab and being a ‘foreign-born male nurse’, and in general nursing being viewed as a feminine, low-status profession. Only two strategies, accessing support from family and other student mothers, used by women to cope with challenges, were identified. Supportive interventions considering gender were limited; these included matching students with support services' personnel by sex, involving male family members in admission and orientation processes, and using patient simulation as a method to prepare students for care-provision of patients of the opposite-sex.

**Conclusion:**

Future work in nursing higher education, especially regarding supportive interventions, needs to address the intersections of gender, gender identity/sexual orientation and foreign-born status, and also consider the complexity of migrant students’ contexts.

**Supplementary Information:**

The online version contains supplementary material available at 10.1186/s12912-021-00678-0.

## Background

In 2017, there were over 5 million international students worldwide (i.e., individuals pursuing educational activities in a country that is different than their country of residence) and this number is increasing annually [1]. This is largely due to a growing demand from students for higher education (college/vocational and university degrees) and the limited capacity in certain countries to meet this need. International experience is also highly valued by many employers and thus studying abroad makes new graduates more competitive in the workforce [2, 3]. On the pull-side, academic institutions are wanting to draw the most talented candidates and are looking to increase their student enrollment and revenues [2, 3]. Most international students are from Asia, in particular China, India, South Korea and Middle Eastern countries, while top destinations for these students are the US, the UK, France, Australia, Canada and Germany [3]. These same countries are also primary resettlement sites, and have substantial numbers of migrants (e.g., immigrants, refugees), especially from low and middle-income countries, enrolled in their colleges and universities [3–7]. This is driven by migrants who desire, or who are required to supplement their previous education in order to integrate into the local workforce, and by the expectations of many migrants for their children (including the 1.5 generation) to obtain an academic degree. Academic institutions in these major host countries are therefore needing to respond to and serve a more diverse student clientele.

Nursing is one of the many disciplines with an increasing number of foreign-born students. There are several benefits to the globalization of nursing education, including strengthening the healthcare workforce capacity (front-line workers, administrators, policy-makers, academics as well as researchers), increasing the linguistic and cultural diversity of nursing professionals, and the sharing of new ideas across countries toward the improvement of nursing practice [8, 9]. Increasing the level of education among nurses also improves health outcomes, enhances gender equality and contributes to economic growth, especially in low-and-middle-income countries [10, 11]. The course of study and clinical training in academic nursing programs however, are demanding and can affect the well-being of students and result in mental health problems [12–16]. Stress in turn can result in failure or students deciding to withdraw from their studies.

The stresses experienced by foreign-born nursing students are magnified due to factors related to their international/migrant status [17–20]. Challenges associated with living in a new country, including financial concerns, discrimination (perceived or actual), adapting to a new culture and language, loss of social support and unfamiliarity with the education, health and other systems, may affect education experiences and compound psychological distress. The challenges experienced and impacts may be patterned by gender. Gender is defined as the ‘socially constructed roles, behaviors, activities and attributes that a given society considers appropriate for men, women, boys and girls’ [21]. The migration process itself is influenced by gender as the opportunity and the level of control over the decision to migrate typically differs between men and women. Fear of being persecuted because of one’s ‘gender identity’ (i.e., a person’s individual experience of gender, which may or may not correspond to one’s biological sex) [22], may also be the reason one decides to migrate. Transit and post-migration experiences also diverge along gender lines, for example risks for gender-based violence, perceptions by the receiving-country society and integration outcomes often vary between male and female migrants and also by sexual orientation or gender identity (e.g., if one identifies as lesbian, gay, bisexual, transgender and/or intersex) [23]. Moreover, international female students compared to male students, have reported facing greater expectations to balance home/childcare responsibilities [24, 25], experiencing more value conflicts regarding gender roles [26, 27], and having stronger emotional and physiological reactions to stress [28, 29]. In contrast, male students have expressed feeling stress associated with social status loss and due to traditional expectations to financially provide for the family, and they have been shown to be more likely to process their stress in solitude [30]. Gender norms can also affect both male and female students’ abilities to relate to members of the opposite sex in academic and clinical settings [27, 31]. To effectively support and promote the success of foreign-born nursing students, academic institutions should therefore ensure that approaches and resources not only take into account the foreign-born context, but also consider the gender dynamics that are shaping students’ experiences.

There is an extensive body of literature on foreign-born nursing students [17, 32–34], however, we did not identify any review that assessed the literature with a gender lens. Within the nursing education literature, reviews that have examined gender have primarily focused on the experiences of male students in general without any mention of a migrant or international background [35–39]; more recent reviews have considered the experiences of nursing students with diverse sexual and gender identities, although the research in this area remains scarce and also does not refer to foreign-born students [40–42]. In parallel, other literature has reviewed or discussed the intersection of gender or gender identity/sexual orientation and international status in relation to students’ experiences and its implications for academic institutions and educators, but none of these address the context of nursing or other healthcare professional education [43–45]. We therefore conducted a scoping review to address this gap. The objective of this scoping review was to map the literature on the challenges, coping responses and supportive interventions for international and migrant nursing students in academic institutions in major host countries with a gender lens.

## Methods

A scoping review is commonly used to explore and summarize what is known on a particular topic [46]. This methodology was therefore selected since our goal was to describe what is known about gender and foreign-born nursing students’ experiences and supportive interventions across a broad array of existing literature while applying a gender lens. We used the Joanna Briggs Institute (JBI) methodology for scoping reviews to guide our approach [46].

### Search strategy

We consulted a university librarian to assist us in selecting the databases and in developing the search strategies. We searched 10 electronic databases including CINAHL, Embase, Cochrane, Medline, Web of Science, the Joanna-Briggs institute EBP database, Psych-Info, Eric, Sociological abstracts and ProQuest. Search terms (subject headings/descriptors, keywords) included those related to international and migrant students and to nursing education; the strategy was adapted for each database and the AND/OR Boolean operators were applied accordingly. Keywords were searched in the titles, abstracts, keywords and subject fields. No language or time restrictions were applied. In order to refine the searches and adjust them for the various platforms, we first conducted test searches in two databases (CINAHL and Medline). An example of one of the search strategies (CINAHL) is presented in Table 1. Additional papers were identified through the examination of the reference lists of literature review papers that met the inclusion criteria.

**Table 1:** CINAHL search strategy^a^

1	(MH “Students, Nursing+”) OR (MH “Students, Nursing, Practical”) OR (MH “Education, Nursing+”) OR (MH “Schools, Nursing”) OR (MH “Faculty, Nursing”)
2	(MH “Faculty-Student Relations”) OR (MH “Education, Clinical+”) OR (MH “Learning Environment+”)
3	Nurs^*^
4	2 AND 3
5	(Nurs^*^ N4 (student^*^ OR education))
6	1 OR 4 OR 5
7	(MH “Students, Foreign”) OR (MH “Transients and Migrants”) OR (MH “Emigration and Immigration”) OR (MH “Refugees”) OR (MH “Immigrants+”) OR (MH “English as a Second Language”)
8	“Born abroad” OR Foreign^*^ OR Immigra^*^ OR Refugee^*^ OR Migra^*^ OR ((International OR minorit^*^) N3 student^*^) OR ((Second OR additional OR proficiency OR native OR nonnative OR primary OR minorit^*^ OR first) N3 language) OR (mother^*^ N3 tongue)
9	7 OR 8
10	6 AND 9

^a^ Lines 3, 5 and 8, are keyword searches that were executed in the following fields: TI (title), AB (Abstract) and MW (Word in Subject Heading)

### Literature selection

We included peer-reviewed research (qualitative, quantitative or mixed methods), discussion papers and literature reviews. Study protocols, abstracts, books and dissertations/theses were excluded. English, French, and Spanish publications were considered. Literature was included if it discussed or reported on challenges, coping responses and/or supportive interventions for foreign-born students studying in an academic nursing program in Canada, the US, Australia, New Zealand or a European country (i.e., high-income countries according to the Organisation for Economic Co-operation and Development that receive large numbers of migrants and international students and that have similar sociocultural norms and political systems) [47]. Challenges were defined as any difficulties experienced by the students; coping responses referred to any strategies that were used by the students to help overcome, minimize or tolerate challenges; while supportive interventions were policies, programs, or strategies meant to address challenges, enhance coping and improve students’ overall experiences. Challenges, coping and/or interventions could have been examined from the perspective of students and/or educators and administrators or could have just been described and discussed generally. Papers that reported on the evaluation or testing of an intervention were also included.

‘International students’ were defined as individuals with student visas but excluding exchange students and those completing only part of their degree abroad. ‘Migrant students’ were defined as individuals born in another country who moved with the intention of resettling in the new country; this includes immigrants, refugees, and asylum-seekers (i.e., individuals in the process of making a refugee claim) who could have migrated as children or as adults (second generation migrants were excluded). We included literature that focused on ‘English-as a second/additional-language’ (ESL/EAL) students without specifying the countries of origin, since foreign-born students often comprise a significant proportion of ESL/EAL students. Papers that focused on ‘minority’ or non-traditional nursing students were also kept if foreign-born or ESL/EAL students were clearly included and there were results and/or implications specific to this population. Similarly, if a paper included or discussed nursing students generally, it was retained if there were study results and/or implications relevant to foreign-born or ESL/EAL students. Literature that included internationally-trained nurses was considered if the nurses were studying in an academic nursing program; we excluded papers that examined internationally-trained nurses who were completing a transition/integration program.

Lastly, ‘Academic nursing program’ was defined as any program leading to a post-secondary degree including college/vocational, bachelor and graduate degrees in nursing. Papers that studied or discussed students from other healthcare disciplines were only kept if there were results and/or implications that referred to nursing students. Papers could have pertained to students in the context of clinical, theoretical and/or research education and training.

**Fig. 1:**
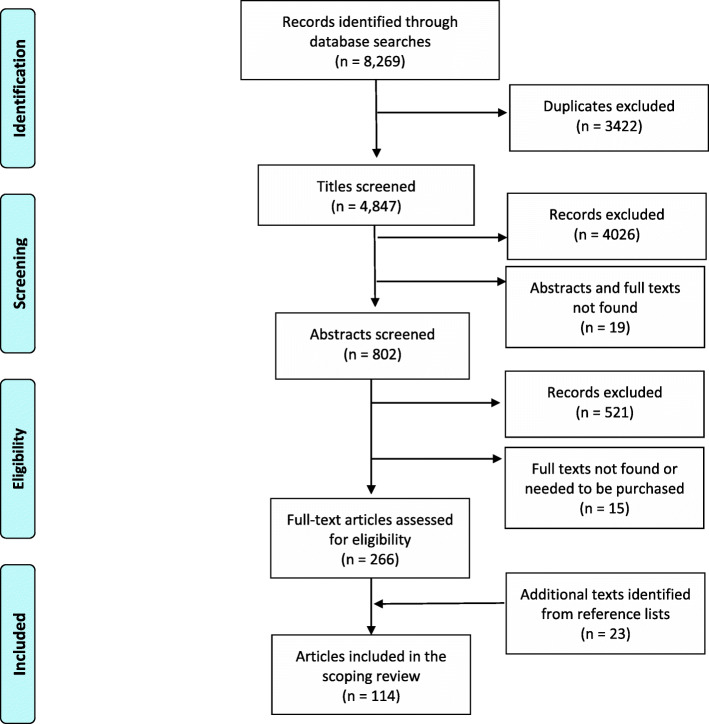
PRISMA Flow diagram. Moher D, Liberati A, Tetzlaff J, Altman DG, Prisma Group. Preferred reporting items for systematic reviews and meta-analyses: the PRISMA statement. PLoS medicine. 2009 Jul 21;6 (7):e1000097

The database searches yielded 8269 records (see Additional file 1 for the search results by database). All citations were downloaded and managed using Endnote. We first removed duplicates and then screened titles to remove citations that clearly did not meet the inclusion/exclusion criteria. We then reviewed abstracts to further eliminate papers that did not meet all of the criteria. For the remaining citations we retrieved and reviewed the full-texts (*n* = 266) in order to confirm eligibility. The screening and selection process was led by KPV and supported by LM and BV via ongoing discussions to ensure that the criteria were being correctly and consistently applied. Articles at this step were mainly excluded because they did not have results and/or implications specific to foreign-born/ESL/EAL students or to nursing students (i.e., all healthcare professionals were examined and discussed together), or because they were theses/dissertations or descriptions of nursing programs that were intended to be advertisements to recruit new students. When there was uncertainty regarding the eligibility of an article, LM independently reviewed it and a decision on whether to include it was made through joint discussion with the other authors. Twenty-three additional papers were identified by examining the reference lists of included review papers. LM read all of the papers and confirmed the final selection (see Fig. 1 for the PRISMA flow diagram).

### Data extraction, analysis and synthesis

For all eligible papers, we extracted and stored data in an excel file including: 1) paper characteristics (publication type, year, and language); and 2) study/review/discussion paper information. For the latter this included the paper objective, the location(s) of the study/discussion/review, the foreign-born student population(s) of interest in the paper (international students and/or migrants and their countries/regions of origin and length of time in the country; for migrants we also sought information on immigration status), the educational context, whether or not the perspectives of educators and/or administrators were considered/discussed, and information on challenges, coping responses and supportive interventions. For studies, we also extracted information on the research design and data collection methods, and for reviews, we recorded the type of review conducted, the number and type of sources (e.g., articles, books), and the process used to identify sources.

To address the review objective, we critically assessed each paper and recorded information related to gender. To do this, we drew on existing frameworks used to conduct gender analyses in health research [48, 49] and LM and BV developed key questions to help guide the assessment. These included the following:

- Was sex included or addressed by the authors/researchers?
- Was gender explicitly considered by the authors/researchers through use of a framework or lens?
- Was gender identity/sexual orientation included or addressed by the authors/researchers?
- Was sex and/or gender considered as a variable in analyses?
- Were findings and/or implications reported separately by sex and/or gender?
- Based on the results and/or discussion points of the papers:
  - Did sex or gender (appear to) play a role in the challenges experienced by students? For example, at the intersection of sex and gender such as roles within the family, cultural/religious conventions that dictate how men and women should behave, differential access to resources, and experiences of discrimination.
  - Did coping responses (appear to) differ by sex or gender?
  - Did interventions (appear to) consider gender roles, norms and expectations?
  - Did interventions (appear to) consider diversity in gender identities/sexual orientations?

KPV was responsible for extracting the paper characteristics and information; LM verified all data extraction. The assessment of papers for gender related information was conducted by two research assistants. To ensure consistency in the process, 20 papers were reviewed by both research assistants. LM independently assessed all papers. All information collected was collated and synthesized into summary tables and text.

## Results

One hundred and fourteen articles were included in the scoping review. A summary of the literature is reported in Table 2. All of the papers were published in English, 12 were discussion papers, 20 were reviews and 82 were research studies. The publication period spanned 39 years (1981–2019) and just over a quarter of papers (*n* = 30, 26%) were published within the last 5 years. Two-thirds of the research were qualitative studies.

**Table 2:** Summary of the literature

#	1st Author (year)	Objective	Methodology^**a**^/Discussion paper/Review type^**b**^	Country^**c**^	Foreign-born Students’ description^**d**^	Methods^**e**^ (or N/A)	Educational context^**f**^
***Research***							
1	Abu-Arab (2015) [50]	To present and discuss the challenges faced by a group of clinical educators in teaching and assessing nursing students from culturally-and-linguistically diverse (CALD) backgrounds in Australian English-speaking hospitals.	Qualitative descriptive	Australia	International students Migrants Creole, Mandarin, Khmer, Malay, French, Korean, Cantonese, Vietnamese, Swahili, Malayalam speaking	8 clinical educators 19 students Questionnaire	Bachelor Clinical
2	Abu-Saad (1981) [51]	To assess the difficulties foreign nursing students encounter in their adjustment to university nursing programs and to evaluate the mechanisms that facilitate their adaptation to university nursing programs.	Quantitative survey with open-ended questions	United States	Foreign-born Asia, Latin America, North America, Middle East, Africa, Western Europe, Scandinavia, South Pacific	82 students Questionnaire	Bachelor Graduate (Masters) Graduate (Doctorate) Clinical
3	Abu-Saad (1982) [52]	To examine actual and potential factors that help Asian students adjust to the nursing program and to describe difficulties encountered.	Quantitative survey with open-ended questions	United States	Foreign-born Asian	Students (sample not specified) Questionnaire	College/vocational Bachelor Graduate (Masters) Graduate (Doctorate) Clinical
4	Abu-Saad (1982) [53]	To examine actual and potential factors that help Middle Eastern students adjust to the nursing program and to describe difficulties encountered.	Quantitative survey with open-ended questions	United States	Foreign-born Iran, Egypt, Lebanon, Jordan, Syria, Israel (Arab only) LOT: average of 4 years	Students (sample not specified) Questionnaire	Not specified
5	Abu-Saad (1982) [54]	To examine whether academic nursing programs in the United States meet foreign nursing students’ and their countries’ needs and expectations.	Quantitative survey with open-ended questions	United States	Foreign-born Asia, Latin America, North America, Middle East, Africa, Western Europe, Scandinavia, South Pacific LOT: 64% < 6 years	82 students Questionnaire	Bachelor Graduate (Masters) Graduate (Doctorate) Clinical
6	Alexander (1991) [55]	To examine the concerns of international students as they face life in a new culture and struggle with a second language, to examine their coping methods and to identify ways that can facilitate their learning.	Ethnography	United States	International students Africa, others not specified	16 students Interviews	Bachelor
7	Ali Zeilani (2011) [56]	To explore the doctoral study experiences of Jordanian students who completed their nursing doctoral degree in the United Kingdom.	Qualitative descriptive	United Kingdom	International students Jordan	16 students Interviews	Graduate (Doctorate)
8	Bosher (2002) [57]	To report the findings of a needs analysis conducted to determine why many English-as-a-second language (ESL) students enrolled in the Associate of Science degree nursing program were not succeeding academically and to report on the development, implementation and evaluation of a course created to respond to students’ challenges.	Descriptive (qualitative and quantitative data)	United States	Migrants Needs assessment: West Africa, East Africa, South East Asia, Caribbean, Former Soviet Union LOT: an average of 5 years Course participants: Liberia, Somalia, Ethiopia, Cameroon, Vietnam, Cambodia, Laos (Hmong), Nepal (Tibetan), China, Haiti, Cuba, Russia, Ukraine, India, Morocco LOT: an average of 5 years; two students 20 or more years	1 program director 5 faculty members 28 students (participated in the needs assessment) 18 students (participated in and evaluated the course) Interviews Questionnaires Observations	College/vocational Clinical
9	Bosher (2008) [58]	To determine the effects of linguistic modification on ESL students’ comprehension of nursing course test items.	Qualitative descriptive	United States	Migrants India (Tibetan), Malaysia (Malay), Laos (Hmong), Ethiopia (Amharic) LOT: 3–10 years	5 students Interviews Group discussions	Bachelor
10	Boughton (2010) [59]	To describe and report findings from an evaluation of a support program for CALD nursing students enrolled in a two-year accelerated Master of Nursing program in Sydney, Australia.	Qualitative descriptive	Australia	Foreign-born Korea, Philippines, Tanzania, United States, Singapore, China, Laos, Romania, Nigeria, Kenya, Zimbabwe LOT: 1 week to 29 years	13 students Interviews	Graduate (Masters) Clinical
11	Brown (2008) [60]	To describe the development, implementation and outcomes of a program to increase the retention and success of foreign-born students challenged with English as a second language at a historically Black university located in Virginia, United States.	Descriptive (qualitative and quantitative data)	United States	Migrants Ghana, Ethiopia, Nigeria, Kenya; Philippines, Vietnam, Mexico, Panama, Caribbean LOT: most > 10 years, two students < 2 years	22 students (provided input for program development) Faculty members (sample not specified) 26 students (outcome data) Focus group Questionnaire Group meetings Interviews Informal discussions University data	College/vocational Bachelor Clinical
12	Cameron (1998) [61]	To report results from an extensive needs analysis for ESL-speaking graduate nursing students with a focus on skills required for school, clinical practice and interaction with a multicultural, socially stratified patient population.	Descriptive/Ethnographic	United States	International students Taiwan, Japan, Thailand, Jordan	16 students (completed tests) 4 division chairpersons in the School of Nursing Clinical preceptors, educators and students (participated in interviews and/or observations, sample not specified) Speaking proficiency test Observations Interviews	Graduate (Masters) Clinical
13	Campbell (2008) [62]	To test the effects of using enhanced language instructions to improve oral and written communication skills for students with limited language proficiency and standard form of instructions.	Pre-test post test	United States	Migrants Chinese, Korean, Haitian, East Indian, Hispanic, Russian	20 students Tests on oral and written performance	College/vocational Clinical
14	Caputi (2006) [63]	To describe how faculty members explored the learning needs of their student population with English-as-an-additional- language (EAL) and offer practical suggestions to help other faculty members.	Qualitative descriptive	United States	Migrants Poland, Romania, Mexico, China, Philippines LOT: 6–18 years	7 students Conversation circles Observations	College/vocational Clinical
15	Carty (1998) [64]	To describe the challenges and support strategies used for Saudi international students in an intensive bachelor of nursing program in Virginia, United States.	Qualitative descriptive	United States	International students Saudi Arabia	12 students Faculty members (sample not specified) Discussions Observations	Bachelor Clinical
16	Carty (2002) [65]	To identify challenges and positive points regarding international nurses’ doctoral education experiences in American schools of nursing.	Descriptive (qualitative and quantitative data)	United States	International students Survey: Taiwan, Thailand, Zimbabwe, Cameroon, Colombia, Iceland, Netherlands, Lebanon, Brazil, Gambia, Greece, Kenya, India, Liberia, Germany, Puerto Rico, Hong Kong, Switzerland, South Korea, China, Japan, Jordan, Canada, Saudi Arabia, Egypt, Jamaica Focus group: Thailand, Egypt, Saudi Arabia	24 universities (presumably administrators and/or faculty completed surveys) 5 students Survey Focus group	Graduate (Doctorate)
17	Carty (2007) [66]	To identify predictors of success of Saudi Arabian students enrolled in an accelerated baccalaureate program leading to a bachelor of science in nursing degree.	Descriptive correlational	United States	International students Saudi Arabia	34 students Student records Application forms	Bachelor
18	Chiang (2009) [67]	To offer additional knowledge and insights regarding teaching and learning barriers encountered by international nursing students and those training them and to describe and report on the evaluation of a transition course developed to support international students at an Australian university’s school of nursing.	Qualitative descriptive	Australia	International students	Students (sample not specified) Educators (sample not specified) Interviews	Bachelor Clinical
19	Colling (1995) [68]	To describe the experiences of international students including how they learn about various nursing schools in the United States, the type of programs in which they enroll, and the barriers they encounter when they come to study and to identify strategies that schools of nursing use to manage the educational and cultural challenges that students face.	Quantitative descriptive	United States	International students Across the schools of nursing: 49 different countries, 50% from Asia 83 students: Asia, western Europe, Canada, Australia, Middle East, Africa, Hispanic countries	83 students 45 schools of nursing Questionnaires	Bachelor Graduate (Masters) Graduate (Doctorate)
20	Crawford (2013) [69]	To report findings from the initial round of interviews of an action research study, in which the project intended to evaluate the English language support program; identify the needs/ perceptions of students in terms of learning needs; and develop appropriate teaching/learning strategies to be implemented.	Qualitative descriptive	Australia	International students Migrants Philippines, Zimbabwe, China, Japan, Egypt, Bangladesh	8 students Interviews	Bachelor Clinical
21	DeBrew (2014) [70]	To describe nurse educators’ experiences where they struggled in their decision to fail or pass a student in clinical, including foreign students and other students with non-traditional backgrounds.	Qualitative descriptive	United States	Foreign-born	24 educators Interviews	College/vocational Bachelor Clinical
22	DeLuca (2005) [71]	To describe what it is like to be a Jordanian graduate student in nursing in the contexts of a new culture, university and realm of professional nursing.	Phenomenology	United States	International students Jordan	7 students Interviews Journals	Graduate (Masters)
23	Donnell (2014) [72]	To examine the associations between English language ability, participation in a reading comprehension program and attrition rates of nursing students in Texas.	Correlational, secondary analysis	United States	ESL students Black, Hispanic/Latino	3258 students (529 were ESL students) Questionnaires	College/vocational Bachelor
24	Donnelly (2009) [73]	To identify factors that influence EAL students’ academic performance from the perspectives of the instructors.	Qualitative descriptive	Canada	Migrants	9 instructors Focus groups	Bachelor Clinical
25	Donnelly (2009) [74]	To gain a greater understanding of how EAL nursing students cope with language barriers and cultural differences and to identify the factors that help or hinder them to succeed.	Mini-ethnography	Canada	Migrants China, Korea, Japan, Romania, Ukraine, Hong Kong LOT: 2.5–10 years	14 students Interviews	Bachelor Clinical
26	Doutrich (2001) [75]	To describe the international educational experiences of Japanese nurses completing a masters’ or doctoral degree in the United States.	Phenomenology	United States	International students Japan	22 students Interviews	Graduate (Masters) Graduate (Doctorate) Clinical
27	Dudas (2018) [76]	To study EAL students’ experience in an accelerated second-degree baccalaureate nursing program.	Phenomenology	United States	International students Migrants Korea, others unknown	12 students Interviews Field-notes	Bachelor
28	Dyson (2005) [77]	To understand the lived experiences of Zimbabwean nursing students and to suggest strategies for improving their educational management.	Life history study	United Kingdom	International students Zimbabwe	9 students 1 nurse Interviews/narratives	College/vocational Clinical
29	Englund (2019) [78]	To investigate the relationship between marginality and nontraditional student status in nursing students enrolled in a baccalaureate nursing program in Texas.	Correlational	United States	ESL students	192 students (32 were ESL) Questionnaire	Bachelor
30	Evans (2007) [79]	To investigate the educational experiences of international doctoral nursing students and their research supervisors.	Qualitative descriptive	United Kingdom	International students East Asia, Middle East	5 students 11 supervisors Questionnaire (open-ended questions)	Graduate (Doctorate)
31	Evans (2011) [80]	To explore the international doctoral student journey; specifically, to investigate the learning experiences of international doctoral nursing students at different points in their journey and to identify best practice in supporting effective learning in this student group.	Qualitative descriptive	United Kingdom	International students European Union, Middle East, East Asia, South Asia, Sub-Saharan Africa	17 students Interviews	Graduate (Doctorate)
32	Fettig (2014) [81]	To explore the role of peer-group interactions in the socialization of non-traditional nursing students in a licensed practical nurses –to-associate registered nurse program in the Midwest, United States.	Qualitative descriptive	United States	International students African countries	10 students Interviews	College/vocational Clinical
33	Gardner (2005) [82]	To gain a greater understanding of the factors that influence foreign-born students’ success in nursing school.	Case study	United States	Foreign-born East Indian LOT: 5 years	3 students Interviews Observations	Bachelor
34	Gardner (2005) [83]	To describe ethnic and racial minority nursing students’ experiences while enrolled in a predominantly White nursing program.	Phenomenology	United States	Foreign-born East Indian, Hispanic, Hmong (Laotian), Nigerian, Filipino, Nepalese, Vietnamese, Chinese LOT: at least 4 years	15 students Interviews	Bachelor
35	Gay (1993) [84]	To describe the international students attending a large school of nursing in the United States, their challenges (from the perspective of faculty members) and the strategies used for dealing with problems.	Case study	United States	International students Finland, Iceland, Japan, Jordan, Korea, China, Taiwan, Saudi Arabia, Thailand	42 students Observations (by faculty)	Graduate (Masters) Graduate (Doctorate)
36	Gilligan (2012) [85]	To: [1] discover the specific needs of CALD students in the Master of Pharmacy, Joint Medical Program and Bachelor of Nursing programs in relation to language and cultural considerations and [2] delineate the attitudes of domestic students to the cultural issues experienced by their peers and patients.	Qualitative descriptive	Australia	International students China, Taiwan, Saudi Arabia, Philippines	35 students (10 nursing students) Focus groups	Bachelor
37	Gorman (1999) [86]	To describe the views and experiences of non-English speaking background nursing students and the faculty members who teach them at two Australian universities.	Qualitative descriptive	Australia	Foreign-born Italy, Russia, Poland, Malawi, China, Iran, Romania, Hong Kong, Singapore, Malta, Vietnam	17 students 14 faculty members Interviews	Bachelor Clinical
38	Greenberg (2013) [87]	To evaluate the effectiveness of a faculty development program on faculty’s self-reported feelings of comfort when acting as an ESL support person, ability to identify their own cultural biases and assumptions, knowledge of barriers and challenges faced by ESL nursing students, and ability to apply the knowledge gained from the project to ESL group sessions.	Pre-test-post-test	United States	ESL students	10 faculty members Questionnaires Observations	College/vocational Clinical
39	Guhde (2003) [88]	To describe and present the evaluation of a tutoring program meant to help ESL students master the English language.	Case study	United States	Foreign-born China	1 student Observations Discussions An evaluation of the student’s ability to understand clinical information	Bachelor Clinical
40	Harvey (2017) [89]	To explore adult international students’ experiences of leaving spouse and children for further education overseas.	Descriptive phenomenology	Australia	International students India, Indonesia, Vietnam, Brunei, the Philippines, Taiwan, China LOT: 2 months- 6 years	10 students Interviews	Graduate (not specified)
41	Havery (2019) [90]	To investigate how clinical facilitators’ pedagogic practices in hospital settings enabled or constrained the learning of students for whom English was an additional language.	Ethnography	Australia	International students Korea, Japan, Cambodia, Taiwan, China, India, Hong Kong, Nepal, Vietnam, Indonesia, Malaysia	21 students 3 clinical facilitators Observations Field-notes	Bachelor Clinical
42	He (2012) [91]	To investigate Chinese international undergraduate nursing students’ acculturative stress and sense of coherence at an Australian university in Sydney.	Quantitative descriptive and correlational	Australia	International students China	119 students Questionnaires	Bachelor
43	Jalili-Grenier (1997) [92]	To 1- determine nursing students’ perceptions of the learning activities which contribute the most to their knowledge and skills; 2- determine students’ perceptions of their learning difficulties; 3- compare the perceptions of ESL and non-ESL students; 4- determine nursing faculty perceptions of ESL students’ learning difficulties; 5- compare the perceptions of ESL students and faculty; and 6- identify needs for educational and/or supportive programs for faculty and students.	Quantitative descriptive	Canada	International students Migrants 21 countries LOT: ages on arrival 1 to 29 years old	179 students 24 faculty Questionnaires	Bachelor Clinical
44	James (2018) [93]	To explore the lived experience of one ethnically diverse nursing student who speaks English as a second language.	Narrative inquiry	United States	Immigrant India LOT: immigrated when she was 11 years old	1 student Informal discussions	Bachelor Graduate (Masters) Clinical
45	Jeong (2011) [94]	To explore the factors that impede or enhance the learning and teaching experiences of CALD students and academic and clinical staff respectively and to identify support structures/systems for students and staff.	Qualitative descriptive	Australia	International students Students enrolled in program: China, South Korea, other countries Participants: China, Philippines, Botswana	11 students 3 clinical facilitators 4 academic staff Focus groups interviews	Bachelor Clinical
46	Junious (2010) [95]	To describe the essence of stress and perceived faculty support as identified by foreign-born students enrolled in a generic baccalaureate degree nursing program.	Interpretive phenomenology with a quantitative component	United States	International students Migrants Nigeria, Cameroon, China, India, Vietnam LOT: < 10 years	10 students Focus groups Interviews Questionnaires	Bachelor Clinical
47	Kayser-Jones (1982) [96]	To identify the facilitating factors that help European and Canadian nursing students’ adjustment to American culture and the university and to describe their learning experiences and difficulties encountered.	Quantitative survey with open-ended questions (qualitative data from open-ended questions were the focus in this paper)	United States	Foreign-born Canada, Norway, Denmark, England, Germany	Students (sample not specified) Questionnaire	Bachelor Graduate (Masters) Graduate (Doctorate) Clinical
48	Kayser-Jones (1982) [97]	To discuss the concept of loneliness and its relationship to the education of foreign nursing students who study in the United States.	Quantitative survey with open-ended questions	United States	International students Asian, Latin American, Canadian, Middle Eastern, African, European, Australian	82 students. Questionnaire	Bachelor Graduate (Masters) Graduate (Doctorate)
49	Keane (1993) [98]	To examine learning styles, learning and study strategies, and specific background variables (primary language, ethnic background and length of time in the United States) in a multicultural and linguistically diverse baccalaureate nursing student population.	Correlational	United States	Foreign-born Nigeria, Ghana, Sierra Leone, Liberia,Dominican Republic, Jamaica, Haiti, Barbados, Nicaragua, Philippines, Hong Kong, Taiwan, China, Europe, Columbia, Peru LOT: 1 to > 10 years	112 students Questionnaires	Bachelor
50	Kelton (2014) [99]	To describe the clinical coach role and present data collected including outcomes achieved when a clinical coach role was implemented to support and develop nursing practice for the marginal performer or ‘at risk’ student.	Quantitative descriptive	Australia	International students ESL students	188 students University student data (outcomes of coaching)	Bachelor Clinical
51	Khawaja (2017) [100]	To examine the relationship between second language anxiety and international nursing student stress.	Correlational	Australia	International students LOT: majority 1–3 years, some < 1 year, others > 3 years	152 students Questionnaires	Bachelor Clinical
52	King (2017) [101]	To explore the perceived effectiveness of standardized patients as a means to achieve academic success among EAL nursing students.	Qualitative descriptive	Canada	ESL students Arabic, Tagalog, Malayalam, Bengali, Afrikaans, other languages- speaking	35 students Focus groups	Bachelor Clinical
53	Leki (2003) [102]	To describe a Chinese undergraduate student’s literacy experiences in her nursing major.	Case study	United States	International students Migrants (participant seems to be an immigrant but paper overall pertains to immigrants and international students) China LOT: 5 years	1 student Students’ professors (sample not specified) Interviews Observations Journals Students’ school documents (e.g., assignments)	Bachelor Clinical
54	Lu (2012) [103]	To elicit clinical tutors’ views on the ways in which EAL nursing students had developed appropriate spoken English for the workplace.	Qualitative descriptive	New Zealand	International students Migrants	4 clinical tutors Interviews	Bachelor Clinical
55	Malu (1998) [104]	To uncover the problems that impeded success for immigrant ESL nursing students.	Case study	United States	Migrants Latin America (region of origin was only mentioned for one student)	Students (sample not specified) Interviews University admission data Observations	College/vocational Clinical
56	Markey (2019) [105]	To explore international student experiences while undertaking Master of Science postgraduate education far from home.	Qualitative descriptive	Ireland	International students Asian	11 students Interviews	Graduate (Masters)
57	Mattila (2010) [106]	To describe international student nurses’ experiences of their clinical practice in the Finnish health care system.	Qualitative descriptive	Finland	International students African, Asian	14 students Interviews	Bachelor Clinical
58	McDermott-Levy (2011) [107]	To describe the experience of female Omani nurses who came to the United States to earn their baccalaureate degree in nursing.	Descriptive phenomenology	United States	International students Oman	12 students Interviews	Bachelor Clinical
59	Memmer (1991) [108]	To identify and describe the various approaches used in baccalaureate nursing programs in California to retain their ESL students.	Descriptive (included qualitative and quantitative data)	United States	Migrants	21 nursing programs (data collected from directors or designees of the programs) Questionnaire	Bachelor Clinical
60	Mikkonen (2017) [109]	To describe international and national students’ perceptions of their clinical learning environment and supervision, and explain the related background factors.	Cross-sectional	Finland	International students Africa, Europe, Asia, North America, South America LOT: 1–33 years	329 students (231 were international students) Questionnaire	Bachelor Clinical
61	Mitchell (2017) [18]	To explore the learning and acculturating experiences of international nursing students studying within a school of nursing and midwifery at one Australian university.	Qualitative	Australia	International students Chinese, others unknown	17 students Interviews Field-notes	Bachelor Graduate (not specified) Clinical
62	Muller (2015) [110]	To present a case study, including an evaluation of a school-based language development and support program for EAL students.	Case study	Australia	International students Asian, others unknown	Students (sample not specified) Faculty and staff (sample not specified) Student data (e.g., number who participated in program, number who accessed resources, fail rates) Faculty and staff feedback through various methods	Bachelor Graduate (not specified) Clinical
63	Mulready-Shick (2013) [111]	To explore the experiences of students who identified as English language learners.	Interpretive phenomenology	United States	Migrants Central America, South America, Africa LOT: came to reside in United States in adolescence or early adulthood	14 students Interviews	College/vocational
64	Newton (2018) [112]	To examine the experiences of registered nurses who supervise undergraduate international nursing students in the clinical setting.	Case study	Australia	International students	6 clinical supervisors Interviews	Bachelor Clinical
65	Oikarainen (2018) [113]	To describe mentors’ competence in mentoring CALD nursing students during clinical placement and identify the factors that affect mentoring.	Cross-sectional	Finland	Migrants	576 clinical mentors Questionnaire	Bachelor Clinical
66	Ooms (2013) [114]	To identify and describe available supports at two universities for non-traditional background students and to measure the students’ perceptions regarding the use and usefulness of these supports.	Cross-sectional with a qualitative component	United Kingdom	ESL students	812 students Questionnaire	Bachelor Clinical
67	Palmer (2019) [115]	To explore the lived experiences of graduate international nursing students enrolled in a graduate nursing program.	Descriptive phenomenology	United States	International students Saudi Arabia, India	12 students Interviews	Graduate (Masters)
68	Rogan (2013) [116]	To describe and evaluate an innovation to assist ESL nursing students at an Australian university develop their clinical communication skills and practice readiness by providing online learning resources, using podcast and vodcast technology, that blend with classroom activities and facilitate flexible and independent learning.	Cross-sectional with a qualitative component	Australia	ESL students Chinese, Korean, Nepalese, Vietnamese, Other	558 students (254 were ESL students) Questionnaire	Bachelor Clinical
69	Sailsman (2018) [117]	To explore the lived experience of ESL nursing students who are engaged in learning online in a Bachelor of nursing program.	Interpretive phenomenology	United States	ESL students Spanish, African, Russian, French, Filipino (Tagalog) speaking countries	10 students Interviews	Bachelor
70	Salamonson (2010) [118]	To evaluate a brief, embedded academic support workshop as a strategy for improving academic writing skills in first-year nursing students with low-to-medium English language proficiency.	Randomized controlled design	Australia	International students Migrants	106 students Student assignment scores	Bachelor
71	San Miguel (2006) [119]	To report on the design, delivery and evaluation of an innovative oral communication skills program (the ‘clinically speaking program’) for first year students from non-English speaking backgrounds in a Bachelor or nursing degree at an Australian university.	Descriptive (included qualitative and quantitative data)	United States	Foreign-born China, Hong Kong, Korea, Vietnam LOT: arrived within the previous 4 years	15 Students 3 clinical facilitators Survey Students’ clinical grades Focus groups Students’ and facilitators’ comments	Bachelor Clinical
72	San Miguel (2009) [120]	To report on an evaluation of the long-term effects of a language program that aimed to improve students’ spoken communication on clinical placements.	Qualitative descriptive interpretive	United States	International students China, Vietnam, Taiwan, Hong Kong	10 students Interviews	Bachelor Clinical
73	Sanner (2002) [121]	To explore the perceptions and experiences of international students in a baccalaureate nursing program.	Qualitative descriptive	United States	International students Nigeria LOT: 5–20 years	8 students Interviews	Bachelor Clinical
74	Sanner (2008) [122]	To describe the experiences of ESL students in a baccalaureate nursing program to develop a better understanding of the reasons for their course failures.	Qualitative descriptive	United States	Migrants Liberia, Philippines LOT: 13–24 years	3 students Interviews	Bachelor Clinical
75	Shakya (2000) [123]	To explore the experiences of a small number of ESL/international nursing students during one year of their studies at a large Australian university.	Hermeneutic phenomenology	Australia	International students Migrants Vietnam, Ethiopia, Iran, Nepal, Philippines, South Africa LOT: 4 months to 10 years	9 students Interviews	Bachelor Clinical
76	Shaw (2015) [124]	To identify key learning and teaching issues and to implement and evaluate ‘group work’ as a teaching strategy to facilitate international nursing student learning.	Participatory action research (descriptive with quantitative and qualitative data)	Australia	International students Middle-East, South East Asia, Europe, Canada, North America, South America	12 students (planning phase) 14 teaching staff (planning phase) 108 students (31 were international students; evaluation survey) Interviews Questionnaire (also included open-ended questions)	Bachelor
77	Starkey (2015) [125]	To explore the critical factors that influence faculty attitudes and perceptions of teaching ESL students.	Grounded theory	United States	ESL students	16 educators Interviews Focus group	College/vocational Bachelor Graduate (Masters) Clinical
78	Valen-Sendstad Skisland (2018) [126]	To shed light on practice supervisors’ experiences of supervising minority language nursing students in a hospital context.	Qualitative descriptive	Norway	Foreign-born	10 Clinical supervisors Interviews	Bachelor Clinical
79	Vardaman (2016) [127]	To describe the transitions and lived experiences of international nursing students in the United States.	Descriptive phenomenology	United States	International students Vietnam, China, Nepal, South Korea, Colombia, St. Lucia, Rwanda, Nigeria LOT: 9 months to 5 years, average of 4.3 years	10 students Interviews	College/vocational Bachelor Clinical
80	Wang (1995) [128]	To describe the experience of Chinese nurses studying abroad.	Phenomenology	United States	International students Taiwan	23 students Interviews	Bachelor Graduate (Masters) Graduate (Doctorate) Clinical
81	Wang (2008) [129]	To describe the experiences of Taiwanese baccalaureate and graduate nursing students studying at Australian universities.	Qualitative descriptive	Australia	International students Taiwan LOT: < 1 year to > 2 years	21 students Interviews	Bachelor Graduate (Masters) Clinical
82	Wolf (2019) [130]	To explore the experiences of Chinese nurses when completing a graduate nursing degree taught in English (as a second language) in the United States.	Case study (included qualitative and quantitative data)	United States	International students China	8 students Survey Interviews	Graduate (Masters) Clinical
***Discussion papers***							
83	Abriam-Yago (1999) [131]	To discuss and present the Cummins Model as a framework for nursing faculty to develop educational support that meets the learning needs of ESL students.	Discussion paper	United States	Migrants	N/A	Any program Clinical
84	Choi (2016) [132]	To provide an overview of the establishment and implementation of a proactive nursing support program purposely designed to address the challenges faced by EAL students.	Discussion paper	Canada	ESL students	N/A	Bachelor Clinical
85	Coffey (2006) [133]	To describe a bachelor of Science in Nursing Bridging Program which aims to address barriers and provide access to employment for internationally educated nurses who are residents in Ontario, Canada.	Discussion paper	Canada	Migrants	N/A	Bachelor Clinical
86	Colosimo (2006) [134]	To discuss how shame affects the learning and experiences of ESL students and present the implications for nursing education.	Discussion paper	United States	International students Migrants	N/A	College/vocational Bachelor
87	Genovese (2015) [135]	To describe the current complexities associated with the process of admitting international students to graduate nursing programs and how to avoid some pitfalls.	Discussion paper	United States	International students	N/A	Graduate (Masters) Graduate (Doctorate) Clinical
88	Henderson (2016) [136]	To provide tips on how to support international students to overcome challenges while studying nursing in Australia.	Discussion paper	Australia	International students	N/A	Bachelor
89	Malu (2001) [137]	To propose six active learning-based teaching tips for faculty teaching ESL students.	Discussion paper	United States	Migrants	N/A	College/vocational Bachelor Clinical
90	Robinson (2006) [138]	To describe the development and implementation of a partnership and program at an American university for foreign nurses from India to obtain graduate education.	Discussion paper	United States	International students India	N/A	Graduate (Masters) Clinical
91	Ryan (1998) [139]	To describe the challenges and strategies used in a program at an American university that provides nurses from Taiwan to obtain a bachelor of science degree in nursing.	Discussion paper	United States	International students Taiwan	N/A	Bachelor Clinical
92	Shearer (1989) [140]	To provide suggestions for teachers who are presented with the challenge of teaching students that use English as a second language.	Discussion paper	United States	International students	N/A	College/vocational Bachelor
93	Terada (2012) [9]	To describe the requirements for admission and the challenges that international and ESL students face while studying in advanced practice nursing programs in the United States.	Discussion paper	United States	International students	N/A	Graduate (Masters) Clinical
94	Thompson (2012) [141]	To explore cultural differences in communication and to identify strategies to improve the experience of international and ESL students studying in advanced practice nursing programs in the United States.	Discussion paper	United States	International students	N/A	Graduate (Masters) Clinical
***Reviews***							
95	Burnard (2005) [142]	To review and discuss some of the research on problems associated with studying overseas and in a different culture and to provide suggestions on how teachers in universities might address these challenges.	Literature review	United Kingdom	Foreign-born	17 sources (books, dissertation, chapters, online material) Medline, library searches and ‘serendipitous findings’	Any program
96	Choi (2005) [143]	To examine the challenges faced by ESL nursing students, and identify strategies and explore the utility of the Cummins model of English language acquisition in educating these students. Recommendations for educating ESL nurses are also made.	Literature review	Canada	ESL students	12 articles Search strategy not specified	College/vocational Bachelor Clinical
97	Crawford (2013) [19]	To discuss the challenges ESL nursing students face in adjusting to Western culture, their difficulties using academic English and technical language of healthcare, and the support programs for these students.	Literature review	Australia	ESL/international students	33 sources (articles and books) Search strategy not specified	Bachelor Graduate (Masters) Clinical
98	Davison (2013) [144]	To investigate the application of mobile technologies to support learning in a specific context, namely nursing education for ‘English as a foreign language’ learners.	Qualitative meta-synthesis	Canada	ESL students	66 sources (articles and dissertations) Databases (ERIC, Education Research Complete, CINAHL)	Not specified Clinical
99	Edgecombe (2013) [145]	To identify factors that may impact international nursing students’ clinical learning with a view to initiating further research on how to work with these students to enhance their learning.	Literature review	Australia	International students	36 articles Databases (CINAHL, ERIC, PubMed, Medline, ProQuest Central, Biomed Central, Joanna Briggs, Cochrane databases, Google Scholar, Sci-Verse-Hub)	Bachelor Clinical
100	Evans (2010) [146]	To review the literature on international doctoral students’ experiences, with specific reference to nursing.	Literature review	United Kingdom	International students	19 sources (book chapter, research report, conference paper, journal articles) Databases (ERIC, CINAHL, PubMed, ASSIA)	Graduate (Doctorate)
101	Gilchrist (2007) [37]	To discuss strategies for attracting and retaining students from diverse backgrounds, including ESL students in nursing education.	Literature review	United States	ESL students	13 articles (other literature related to other student groups who face barriers in nursing education was also included) Search strategy not specified	Bachelor Clinical
102	Greene (2012) [33]	To discuss the barriers to educational success among internationally born students and to propose practical, evidence-based strategies that nursing faculty can implement to help international students succeed in nursing school.	Literature review	United States	International students Migrants	31 sources (articles, books) Search strategy not specified	College/vocational Bachelor Graduate (Masters) Graduate (Doctoral) Clinical
103	Hansen (2012) [147]	To discuss areas of difficulty for ESL nursing students and to recommend strategies that can be employed by supportive faculty to assist these students.	Literature review	United States	ESL students	35 sources (book chapters, articles) Search strategy not specified	College/vocational Clinical
104	Koch (2015) [42]	To identify studies which describe the clinical placement experiences of nursing students who have a broad range of diversity characteristics.	Literature review	Australia	International students Migrants	6 articles (other literature related to other student groups who face barriers in nursing education was also included) Databases (CINAHL, PubMed, Academic Search Complete, Medline, Education Search Complete, Health Source: Nursing/Academic Edition, Science Direct, Scopus, Google Scholar) and reference lists of potentially relevant studies	Bachelor Clinical
105	Kraenzle Schneider (2019) [148]	To discuss the challenges of international doctoral nursing students and recommend strategies to support them.	Literature review	United States	International students	17 articles Databases (CINAHL, Medline, PsychInfo, PubMed, Scopus) and ‘other search methods’	Graduate (Doctorate)
106	Lee (2019) [149]	To examine the effectiveness of programs to improve (clinical) placement outcomes of international students and to collate recommendations made by international students and/or placement supervisors that they felt might improve placement outcomes.	Systematic review	Australia	International students	10 articles (other literature related to other disciplines was also included) Databases (PsychInfo, CINAHL Plus, ProQuest Central, ERIC, Informit A+ Education, Informit MAIS) and reference lists of included articles	Bachelor Graduate (Masters) Clinical
107	Malecha (2012) [17]	To identify and summarize what have been reported as stressors to foreign-born nursing students living and studying in the United States.	Literature review	United States	International students Migrants	11 articles Databases (ERIC, CINAHL, MEDLINE, PsychInfo, Web of Science) and reference lists	College/vocational Bachelor Clinical
108	Mikkonen (2016) [20]	To describe the experiences of CALD healthcare students’ in a clinical environment.	Systematic review of qualitative studies	Finland	International students Migrants	12 articles Databases (CINAHL, Medline, Scopus, Web of Science, Academic Search Premiere, ERIC, Cochrane library) and reference lists of included studies	Bachelor Clinical
109	Newton (2016) [150]	To review the literature reporting on the experiences and perceptions of registered nurses who supervise international nursing students in the clinical and classroom setting.	Integrative literature review	Australia	International students	10 articles Databases (CINAHL, Informit, PubMed, Medline, Journals@Ovid, Findit@flinders)	Bachelor Clinical
110	Olson (2012) [34]	To identify the barriers and discover bridges to ESL nursing student success.	Literature review	United States	International students Migrants	25 articles Databases (Academic Search Premier, CINAHL, PubMed, DAI, ERIC) and reference lists from the first database run	College/vocational Bachelor Clinical
111	Scheele (2011) [151]	To synthesize the existing literature on Asian ESL nursing students including their challenges encountered and academic strategies to help these students.	Systematic review	United States	International students Migrants Asian	15 articles Databases (CINAHL, LexisNexis, Expanded Academic ASAP plus, Medline, Cochrane Database of Systematic Reviews, PsychInfo)	Bachelor Clinical
112	Starr (2009) [152]	To synthesize the current qualitative literature on challenges faced in nursing education for students with EAL.	Meta-ethnographic synthesis of qualitative literature	United States	Migrants	10 articles Databases (CINAHL, ERIC, PubMed, EbscoHost, Medline)	Bachelor Clinical
113	Terwijn (2012) [153]	To synthesize the existing literature on the experiences of international students in undergraduate nursing programs in English-speaking universities.	Systematic review	Australia	International students	19 articles Databases (CINAHL, Medline, EBSCOHost, ERIC, PsychInfo, MedNar, ProQuest Dissertations and Theses, Google Scholar + several others (*n* = 37 total)) and reference lists of suitable articles collected during the search process	Bachelor Clinical
114	Wang (2015) [154]	To report the current knowledge on the Chinese nursing students’ learning at Australian universities.	Narrative literature review	Australia	International students Chinese	15 articles Databases (A+ Education, Australian Bureau of Statistics, CINAHL, ERIC, Medline, ProQuest), table of contents of 14 journals and reference lists of relevant articles	Bachelor Graduate (Masters) Graduate (Doctorate) Clinical

^a^ The methodology is based on what was reported in the paper. If a general qualitative methodology was used, it is described as ‘Qualitative descriptive’

^b^ The review type is based on what was reported in the paper. If no specific type of review was named, it is described as a ‘Literature review’

^c^ For discussion papers, the country is based on the location that was the focus of interest in the discussion. For reviews, the country is based on the country where the first author is based (since almost all reviews included literature from multiple countries and did not focus on a specific country)

^d^ For research papers that included student participants, the description of students indicates whether participants included international students and/or migrants; ‘foreign-born’ is indicated if it’s clear that foreign-born students were included but it’s unclear whether they were international students and/or migrants; ‘ESL students’ is indicated if it was not explicitly stated that foreign-born students were included in the study (and there was no explicit mention of international students and/or immigrant students). Country/region of origin or ethnic/language background and LOT (length of time) in country are indicated if information was available for these indicators. For research papers that included only educators and/or administrators as participants, discussion papers and reviews, the description of students is based on the focus of the paper – i.e., international students and/or migrants or foreign-born or ESL students; country/ethnic background is indicated if a specific group was examined

^e^ For research papers, the methods include the sample (the number of student and/or educator/administrator participants) and the methods used to gather data. For reviews, the methods include the number and type of sources included in the review and the process used for identifying sources

^f^ For research papers, the educational context is based on the degree level of the student participants and/or the degree level of the students who were supervised and educated by the educator participants. ‘College/vocational’ refers to a level of qualification that is between a high school diploma and a bachelor’s degree. For discussion papers and reviews, the educational context is based on the degree level that was the focus of interest in the paper or the degree level that the results pertain to. In all instances, ‘Clinical’ is indicated if the clinical context was examined or discussed in the paper

### Focus of the research, discussion papers and reviews

Twenty-two of the research papers primarily focused on highlighting challenges faced by foreign-born students; nine of these included the perspectives of educators (Table 2). Seventeen research papers aimed to identify or examine coping responses and factors that facilitated success among foreign-born students, while 24 papers generally explored students’ and/or educators’ experiences. Twelve research articles described and reported the findings of evaluations of support programs, courses or other strategies meant to support foreign-born/ESL/EAL students and seven other papers were intervention studies (including qualitative and quantitative), which mostly sought to help students’ overcome learning difficulties due to language barriers.

The discussion papers and reviews had similar foci (Table 2). Three discussion papers provided tips on how educators and institutions can support foreign-born/ESL/EAL students, five discussed challenges, implications and strategies to address these, and four other papers described programs, frameworks or approaches to promote the success of students. Among the 20 review papers, all but three included a mix of qualitative, quantitative and other types of literature and only three specifically named the type of review being conducted. Most (*n* = 12) aimed to synthesize the literature on foreign-born/ESL/EAL students’ challenges and support strategies for these students, while five were reviews of the literature of foreign-born/ESL/EAL students’ general experiences, and two focused on interventions including mobile applications to support ESL students’ learning, and programs to improve clinical placement outcomes of international students.

### Locations, educational contexts and populations

The majority of the research (57%) was conducted in the United States; four studies were conducted in non-English speaking countries (Norway and Finland) (Table 3). All but three of the discussion papers, and one review were also specific to the United States context. Several of the research papers pertained to more than one level of education; overall bachelor or college level studies were included in 90%, and graduate level education in 42%, of studies (Table 3). Four discussion papers were limited to bachelor level, four were focused on graduate level, and four others were relevant to nursing education in general. The literature reviews tended to be non-specific, however one and two papers respectively focused on bachelor and doctorate level education. The clinical learning environment was mentioned in two-thirds of the research papers, although was the primary focus in 18% of the research (Table 3). The clinical context was also the main focus in six of the reviews.

**Table 3:** Characteristics of the research studies

Descriptor	Papers ***N*** = 82, % (n)
Methodology	
Qualitative^a^	67.1% (55)
Quantitative^b^	24.4% (20)
Mixed	8.5% (7)
Location of the study	
United States	57.3% (47)
Europe^c^	12.2% (10)
Australia	24.4% (20)
Canada	4.9% (4)
New Zealand	1.2% (1)
Student group^d^	
International students	46.3% (38)
Migrants	15.9% (13)
International students and migrants	11.0% (9)
Foreign-born non-specified	17.1% (14)
English-as-a-second language students	9.8% (8)
Education level^d,e^	
College/vocational	17.1% (14)
Bachelor	73.2% (60)
Masters	22.0% (18)
Doctorate	15.9% (13)
Graduate (not specified)	3.7% (3)
Clinical learning environment was a primary focus	18.3% (15)
Academic or clinical educator and/or administrator participants	34.1% (28)
Student participants	89.0% (73)
Student participants’ sex	*N* = 73^f^
Males	2.7% (2)
Females	12.3% (9)
Males and Females	56.2% (41)
Not specified	28.8% (21)
Student participants’ region of origin^e^	*N* = 73^f^
North Africa and/or Middle East	31.5% (23)
Sub-Saharan Africa/Africa unspecified and/or South Africa	39.7% (29)
Caribbean	8.2% (6)
Latin America	21.9% (16)
Eastern Europe and/or Russia	9.6% (7)
South Asia	19.2% (14)
South East Asia	39.7% (29)
East Asia	45.2% (33)
Unspecified Asia	26.0% (19)
Western/Northern/Southern Europe, North America	
(excluding Mexico), and/or Australia	17.8% (13)
Unspecified	26.0% (19)

^a^ Two also included some quantitative data

^b^ Seven also included some qualitative data

^c^ Includes the United Kingdom (*n* = 5), Finland (*n* = 3), Ireland (*n* = 1) and Norway (*n* = 1)

^d^ Based on the student participants and/or the focus of the paper

^e^ A study may be counted in more than one category so percentages do not add to 100%

^f^ Based on studies with student participants

Across the literature students were described using different terms including ‘foreign-born’, ‘ESL’, ‘EAL’, ‘culturally-and-linguistically diverse (CALD)’, ‘international students’, ‘non-English-speaking background’, ‘immigrants’, and ‘minority or non-traditional students’; in other instances, students were described based on their ethnic background or origin. Length of time in the host country was generally not highlighted; just over a third (34%) of studies with student participants mentioned some information on length of time. International students were the main population of focus in almost half of the studies (Table 3). Similarly, they were also the main focus in seven discussion papers and eight of the literature reviews. Thirteen studies, three discussion papers and one review focused specifically on migrants. The remaining literature examined a mix of international students and migrants or were non-specific in their description of the student population (i.e., described as foreign-born or ESL students).

For migrant students, migration history or status were not reported in the description of the participants in any of the research papers nor were they mentioned or discussed in the review and discussion papers. There were five studies however, that implied based on other sections of the paper that they may have included student participants with a refugee or difficult migration background (i.e., political unrest in their country) [57, 77, 84, 104]. Only one research paper explicitly mentioned students with a refugee background in the introduction and discussion sections [57].

In the research studies with student participants (*n* = 73), students were mainly from East Asia, Sub-Saharan Africa and South East Asia; top source countries in descending order, were China, Vietnam, the Philippines, Korea, India and Taiwan. Asian students (Taiwan = 1, India = 1, China = 1, and one unspecified) were also the population of interest in two discussion papers and two reviews. Instructors/educators were participants in 34% of studies (Table 3) and their perspectives were also explicitly mentioned in two of the literature reviews.

### General overview of challenges, coping responses and supportive interventions

Language and communication barriers, including oral and written expression and comprehension, were the challenges highlighted most often in the literature [9, 17–20, 33, 34, 37, 42, 50–53, 55, 57–65, 67–76, 78–113, 115, 117–123, 125–131, 133, 134, 136, 139, 141–143, 145–148, 150–154]. Language and communication issues occur in academic and clinical settings as well as in social contexts. Learning nursing and medical terminology and colloquial expressions and adapting to a ‘low context communication’ style, were noted as particularly difficult. At the graduate level, academic writing was the major issue, including demonstrating critical analysis [71, 79, 80, 115, 130, 146, 148].

Cultural barriers were also frequently noted [9, 17–20, 34, 42, 51–56, 59, 63, 64, 67, 68, 71, 73–77, 79, 80, 82–87, 89, 96, 97, 100–103, 105–107, 111, 112, 115, 117, 119, 120, 122, 124, 125, 127, 129, 132, 136, 138, 139, 142, 143, 145, 147, 148, 150, 152–154]. These included, for example, divergent views regarding the role of nurses in patient care, and different styles of relating socially whether it be with friends or in care-provider-patient interactions. Difficulties with the supervisory-graduate student relationship were identified as well, as international students often expect structured guidance and for supervisors to be readily available to them based on the supervisory styles they have observed in their home countries [56, 80, 146]. The most apparent cultural challenges described were in the classroom milieu; a number of papers reported that foreign-born students struggle with ‘Western’ learning, teaching and evaluation methods (e.g., self-directed and interactive learning, critical analysis and debating). Self-guided learning and conducting independent research were particular concerns for doctoral students [56, 146]. All these issues are due to the fact that many foreign-born students come from cultures where teaching is primarily didactic, rote learning is encouraged and students are expected to be passive and to not question instructors. Educators and clinical preceptors are equally challenged in this dynamic and feel unable to assess whether students have understood content and instructions, especially when language barriers are significant [65, 74, 79, 94, 112, 126, 150]. In the clinical context this also raises concerns about patient safety [50, 74, 99, 112, 150]. Overall, educators/supervisors and preceptors expressed feeling that they have insufficient time and resources to adequately support foreign-born students [65, 74, 79, 94, 112, 126, 150].

In addition to cultural issues, foreign-born students also struggle with the unfamiliarity of the healthcare system and clinical setting [9, 19, 20, 37, 42, 65, 92, 96, 99, 100, 102, 112, 115, 129, 132, 146]. For graduate students, often they are unable to work clinically in the receiving-country and so they grapple in making links between the theory/research and practice. Regardless of the education level, for students who return to their home country post-graduation, the course content and skills learned, and for graduate students, the research conducted, are not always relevant and applicable to their context [53, 54, 75, 84, 85, 129, 148]. Conducting research internationally is also not always feasible due to a lack of funding and/or supervisory support abroad [148].

Other challenges experienced by foreign-born students included loneliness, social exclusion/isolation, discrimination, resettlement issues (e.g., immigration, housing), financial concerns and maintaining a work-life balance [9, 17–20, 33, 34, 37, 42, 52, 53, 55, 59, 65, 68, 71, 73–75, 77, 79, 80, 82–84, 87, 89, 91, 93–97, 100, 103, 105–107, 109, 111, 113, 115, 120–123, 127–129, 138, 139, 142, 143, 145–148, 150, 152–154]. Access to research funding, limited interaction with student peers and transitioning from a leadership role (held in their home country) to a student position, were challenges specifically noted by international graduate students [79, 80, 148]. Feeling inadequately prepared or overwhelmed and unable to optimize their skills upon return to the home country, were also highlighted as particular issues at the graduate level [56, 75, 79, 148]. Mental health problems, including stress, feeling pressure to succeed, depression, a loss of self-esteem, feelings of guilt (for leaving their families) and anxiety, were commonly reported across the literature irrespective of the level of education [9, 17–20, 33, 55, 65, 68, 71, 75, 76, 79, 80, 82, 84, 86, 89, 91, 93–95, 97, 98, 100, 102, 105, 106, 111, 112, 115, 117, 122, 126–128, 130, 134, 138, 139, 142, 143, 145, 146, 148, 152–154].

The main coping responses used by foreign-born nursing students to overcome challenges, included accessing support (emotional, practical and/or informational) from family and friends, especially student peers with a similar cultural or linguistic background, and staying focused and determined to succeed [18, 20, 34, 52–54, 66, 73, 76, 79–82, 89, 91, 93–96, 103, 105–107, 111, 115, 117, 121–123, 127–130, 141–143, 145, 146, 148, 151–154]. Maintaining their culture and values, but also accepting and being open to differences, were identified as coping mechanisms to deal with cultural barriers, while positive thinking and celebrating successes, were highlighted as ways that students boost their sense of self-worth and reduce stress [20, 34, 56, 64, 71, 73, 79, 80, 85, 89, 91, 93, 96, 107, 123, 127, 145, 152–154]. Numerous papers also reported that students use various strategies (e.g., asking for clarifications, using non-verbal communication, doing additional reading), and actively develop their skills, in order to gain confidence and overcome language and academic barriers [18, 20, 61, 71, 73, 75, 76, 80, 82, 84, 88, 90, 92, 93, 98, 99, 101, 103–107, 109, 111, 113, 115, 117, 120, 123, 127–130, 133, 146, 151–153].

There were several interventions that were described or suggested in the literature as being potentially helpful to foreign-born students (reported in Table 4); the vast majority of these were based on anecdotal evidence. At the structural level, it was recommended that institutions be actively committed, in the form of mission statements, action plans and dedicated resources, to cultivating an inclusive and equitable education environment [17, 20, 33, 37, 50, 51, 53, 63, 64, 68, 74, 79, 80, 82, 92, 94–96, 103, 106, 108, 110, 112, 113, 115, 117, 119–121, 125–127, 129, 132, 138, 139, 141, 145–154]. Equally noted was the importance of promoting diversity and fostering a sense of belonging [17, 37, 108, 127, 145, 146, 148, 151–154]. It was also recommended that educators and preceptors receive training to ensure that they are aware of the challenges that many foreign-born students encounter and to provide them strategies and tools for teaching a multi-lingual and culturally diverse student population [17, 18, 33, 34, 37, 50, 60, 63–65, 68, 71, 73–85, 87–89, 91–96, 103, 106–108, 111–113, 115, 117, 120, 121, 123, 125–128, 131, 138, 139, 141, 143, 145–147, 149–154]. It was also suggested that instructors have smaller classes, clinical groups and student-supervisory ratios, and more time allotted to devote to foreign-born students [20, 50, 51, 64, 65, 74, 79, 104, 108, 112, 120, 121, 127, 129, 139, 147, 149–151, 153].

**Table 4:** Summary of supportive interventions^a^

***Institutional level policies and general support***	
Hold a pre-admission meeting or interview or request a taped personal statement	
Accept a group of students from the same country as a cohort	
Provide information pre-departure (what to expect, what to bring...) / have a dedicated website	
Initiate mentorship/advisor relationship prior to arrival	
Have a more intensive screening process to identify students who will require additional support / develop and apply strict criteria (language, academic...) for acceptance (especially if support for students with language barriers is limited)	
Have an orientation at the beginning of the program (include social and cultural sensitization, raise their awareness to challenges that they will face, provide information about available resources)/provide ongoing information sessions throughout the program	
Send information letters and/or invite students’ families to orientation to inform them of intensity of the program	
Create a handbook, fact sheet or brochures with information about the program, expectations and practical information	
Provide practical assistance with resettling (administrative and immigration support, finding lodging, healthcare, and transportation etc.) / link students with a host family / provide living accommodations / refer to cultural community supports	
Offer childcare for students with children	
Provide financial assistance or scholarships/ inform and support students’ applications for scholarships and studentships	
Create and encourage work or volunteering opportunities (in a healthcare setting, research) /offer work-study initiatives	
Support applications for a range of different research funding (international funding sources)	
Have a designated liaison person or persons for migrant and international students (a paid position or faculty member with release time)(who speaks the students’ maternal languages)	
Have student advisors who meet regularly one on one with students	
Offer courses and services for language training (prior to beginning the program and ongoing services throughout) / have a dedicated course that is integrated into the program/ create opportunities for practicing inside and outside of the classroom and clinical environment / use other strategies (e.g., intensive language drills, role plays, flash cards with terminology, encourage students to listen to tapes, provide feedback on language pronunciation, spelling of words, encourage students to develop a vocabulary journal, use online resources)/ hire language specialists / monitor language development	
Provide tutoring and academic support services	
Provide workshops / additional courses (e.g., on note taking, to develop technology and computer skills, on test taking, critical thinking, assertiveness and communication, quantitative reasoning, how to participate in study groups, time management, studying, writing and formatting, e.g., APA, publishing, plagiarism)	
Provide writing support (editing/proofreading)	
Pair foreign-born students host country students to practice language / give guidance	
Create and encourage participation in student study groups (with a mix of students)	
Provide a mentorship program with alumni	
Create student / community support groups /buddy program with peers	
Support students to maintain connection with family back home	
Provide counselling/ pastoral services (culturally matched) for discussing problems	
Provide psychological support to promote self-efficacy and empowerment; shift students’ locus of control from external to internal, encourage them to not dwell on small issues and to focus on the positive and successes, and promote students to be active in finding solutions (using resources, seeking support)	
Offer social activities /hold activities that celebrate cultural diversity /have events that include families	
Create associations and organizations on campus for students to get involved in / encourage involvement	
Have student spaces that promote sense of belonging and connection (e.g., student lounge, shared office space) / promote belongingness	
Have a designated prayer time and space in the institution/clinical environment / avoid religious holidays as due dates for assignments and exams	
Implement a strategy and have a mission statement and designated resources that promote inclusion and diversity	
Increase the diversity of the student and faculty body (including clinical instructors), especially as role models	
Use a newsletter and other modes of communication to give visibility on international/migrant students, and as medium for communicating information	
Provide training on racism for students and faculty	
Offer courses / learning for all students on cultural diversity / competency /include international placements for students	
Ensure institutional support is available for educators/offer training to academic and clinical educators to raise awareness on students’ challenges and on how to address needs of students /create structures for clinical sites and academic institutions to work closely together to create inclusive and supportive environments/dedicate funding for the creation of structures and resources	
Foster a team approach between colleagues for supporting students / encourage educators to collaborate with support services	
Offer cross-cultural communication workshops and discussions with educators /encourage educators to visit and get to know different communities	
Create a forum where educators and students can meet and exchange regularly on student issues	
Encourage and support educators to visit the countries of origin of students (to raise awareness of care context, care practices and common illnesses; build research network)	
Invite nurse leaders from abroad to come give lectures/ presentations	
Establish and maintain a network with students post- graduation / use network for developing international placements for local students	
Gather data on diversity indicators (use well defined variables to capture specifics, e.g., international students vs. students with English as an additional language) and outcomes	
Provide (additional) support to help students prepare for licensure exams, career planning or more education / provide support to help students integrate post-graduation	
Offer a ‘reintegration’ seminar to support students to deal with conflicts that they may face when they go back to their home country	
Hold a career day (to promote perseverance in the program and to support career planning)	
Survey students to assess their needs / assess students’ satisfaction with services and resources	
***Teaching and research training***	
Require students to take pre-requisite courses before officially starting the program / have a qualifying session (‘visiting student’ status)/ offer transition courses /provide additional time to complete the program / adapt the program	
Offer a flexible course schedule (evenings)	
Adapt courses so that students can maintain a work-life balance	
Offer smaller classes / adjust supervisor-student ratios for graduate supervision (fewer students per supervisor)	
Speak more slowly when giving lectures, structure the content, avoid abbreviations or explain them, avoid idioms, give handouts, provide information in writing, use audio and visual supports when presenting material, review and repeat key elements with opportunity to ask questions and discuss	
Review course content for cultural biases	
Provide students with real situations taken from nursing practice and use storytelling to provide more context/ provide more instruction on the healthcare system / base assignments on clinical experiences / provide experiential learning activities or community projects/ explain culturally bound concepts	
Use a variety of teaching methods / adapt content to be more culturally relevant (e.g., present examples and assignments relevant to the student’s cultural community or country of origin)	
Engage students to share their (cultural) perspective in discussions / foster exchanges and learning between peers	
Organize course content so that students can adapt to the pace and style over time	
Provide more structured support and foster more self-directed learning over time (e.g., review students’ note taking and give feedback, give them guidance on how to identify important information) / make expectations very explicit	
Encourage students to ask questions / invite students to submit questions by writing	
Use clickers in class (provides opportunity to answer questions anonymously)	
Verify students’ understanding	
Challenge students (ask questions to push their thinking)	
Audio record classes (to practice listening to language and review material that may have been missed during class)	
Use concept maps to develop conceptual and language learning	
Use group work to foster peer learning (mix stronger and weaker students/ mix students with different backgrounds and experiences)	
Use oral presentations to develop language skills (in a supportive environment) /let students with language barriers present after other students so that they have a model to work from/ use the pair share approach (let each student present to another student or in small groups and build up to larger groups over time) /allow additional time for preparation of presentations	
Have writing assignments that require personal reflections and opinions / encourage journal writing (to promote writing and expressing own ideas)	
Provide writing examples for assignments	
Provide frequent and detailed feedback to students	
Provide additional time for test-taking (reduce over time as students become progressively stronger in language) / offer a different environment for test-taking (to reduce anxiety)	
Provide an opportunity to practice test-taking	
Ensure tests are written in clear, grammatically correct English (host-country language) / provide synonyms for terms that may not be easily understood / avoid culturally bound language	
Allow dictionaries or translators during tests / encourage the use of dictionaries when writing assignments	
Consider using different evaluation methods/ adjust tests and assignments and make them more complex over time (once students have developed language skills and have adapted to the pedagogical approach) / have more frequent smaller tests / allow more time to complete assignments/ don’t grade initial work, provide feedback and allow opportunity to revise	
Review tests and assignments with students	
Offer to meet with students one on one/ communicate frequently with students / have regular office hours	
Build self-esteem (give positive feedback, promote leadership, focus on strengths, create positive learning experiences, give feedback constructively and confidentially)	
For graduate students take time to discuss research topics and identify an area of focus/ allow students to choose a research focus that is relevant to their country of origin	
Provide research and learning activities according to the student’s needs (e.g., if they plan to return to their country provide activities relevant to the context in which they will work)	
Offer academic (online) resources that are relevant to their country/language	
Provide opportunities for professional development and academic networking (e.g., conferences, student seminars, research groups, research activities like publishing, committees, joining professional organizations)	
Provide leadership training	
***Clinical training***	
Offer information sessions prior to clinical placements including social and cultural aspects / provide a full day orientation with a clinical preceptor / hold an ‘initiation clinical experience’/ discuss issues that may be culturally different or different than nursing in their country (e.g., roles of nurses)	
Offer a community placement experience as an opportunity to build and practice clinical communication (to build language and culture skills)	
Make the clinical environment welcoming (staff, patients)	
Create a network to support clinical learning	
Provide clinical mentors for students / use role modeling	
Adjust the pace and allot additional time to complete clinical training/ provide additional learning opportunities (e.g., practice clinical skills, give more verbal reports, expose students to situations they may not have experienced in their country) / offer an additional course to support clinical learning/provide more hands-on learning	
Have smaller clinical groups / mix groups to foster peer learning / use a team approach to supervise and provide feedback and learning opportunities / assign the same nurse preceptor so a relationship can build over time / ensure adequate release time for nurses providing clinical instruction or supervision	
Provide more verbal and written feedback on clinical performance / debrief regularly with students	
Before students perform clinical tasks verify their understanding using the teach back method	
Provide structured guidance (e.g., provide written protocols, a standardized form to facilitate documentation, and a list of questions for engaging with patients) / speak slowly and repeat during clinical instruction / review clinical documentation and provide detailed feedback	
Be attentive to situations that make students uncomfortable due to cultural differences and adjust activities to allow students time to adapt (to reduce anxiety) / adjust timing of clinical rotation to allow time to develop communication skills first / offer opportunities to build confidence (e.g., match with patients with same language, allow students to demonstrate skills learned from their country origin)	
Ask students to complete a health education assignment whereby they teach something about their country or culture to the clinical staff (to practice teaching and to share their culture and to promote their contribution) / ask students to draw on cultural experiences to gain insight on patient health problems	
Have more assignments that involve clinical documentation	
For students who cannot do clinical placements provide simulation activities	
***Teaching, clinical or research***	
Be supportive and understanding of students’ situation / be respectful / avoid stereotyping /advocate for students	
Make students feel that they are important/know their names and how to pronounce it, ask students how they are doing, ask them their needs, offer assistance and refer to services, take interest and make them feel valued, relate in personal and informal ways	
***Other specific interventions identified in the literature***	
Linguistic modification (simplification of language to ease reading load and to increase comprehension) of written material [34, 58]	
Course for students to enhance assertiveness, communication and information gathering during clinical interactions and to help students understand and navigate cultural differences [57, 143]	
Support program to help with academic, communication and relational challenges (a series of workshops that include interactive delivery, activities in small groups and the use of video clips, reflective feedback sessions, and open discussions; also includes support for faculty) [59]	
English language program to address communication challenges [61]	
Enhanced language instruction (workshops to promote oral and written comprehension and expression) [62]	
A full semester transition course to develop clinical and communication skills and to adapt to the new clinical context [67]	
PowerPoint learning modules for faculty about barriers experienced by students, strategies to increase cultural competency, strategies to help students overcome language barriers, and strategies to promote academic success [87]	
Intensive individual or group tutoring to practice listening, recording, and transmitting clinical information (to overcome language barriers) [88]	
Clinical coaching / a Clinical Communication Programme (a tool to assist students to understand and apply professional clinical language and jargon common to the clinical environment and to be able to document accurately and to be computer literate) [18, 99]	
Standardized patient simulation to foster a supportive and contextually rich environment for nursing student learning (to practice language, to practice skills, to debrief in a safe space) [101]	
Clinically-speaking workshop (an intensive workshop on clinical communication) Clinically speaking online clinical language resources (a podcast which serves as an audiovisual terminology resource; a vodcast which provides examples of models of nursing interactions in typical clinical scenarios) [20, 109, 116, 119, 120, 149, 150]	
An English for specific purposes (ESP) program to develop English proficiency for academic studies and clinical placements: includes a face-to-face course (writing and proofreading, note-taking, colloquial language, speaking skills, medical terminology, clinical assessments and handovers, intensive language training); support to educators in class and for developing teaching and assessment materials and for giving feedback/support to students; online resources (reading, listening and writing, social/cultural, vocabulary, speaking/pronunciation) [110]	
An intensive, embedded academic support workshop [118]	
A scaffolded small group work intervention to enhance learning for both international and domestic students [124]	
English as an Additional Language support program (mentorship, one on one support, referrals, workshops, and social connection) [132]	
A bridging program for internationally trained nurses to integrate into a fast-tracked bachelor/graduate program (focuses on language development, cultural adaptation, exposure to clinical context/job-shadowing, and theoretical knowledge) [133, 138]	
International partnerships / alliances [64, 138, 139]	
Mobile application for language learning and support (to look up terminology, for communication with instructor during clinical, practice exercises, to listen to podcasts for practicing language, a dictionary, and translation software) [144]	

^a^ Based on the results and discussion sections in the research and review articles and based on the reflections/discussion points of authors in the discussion papers

According to the literature it may also be beneficial if students have more time to complete their degree, or if the program is adapted to better suit their needs (e.g., an additional session or qualifying year to take pre-requisites, a transition semester with courses modified to allow students to acclimate to their new environment, and/or extra clinical training) [37, 60, 71, 79, 102–104, 108, 126, 133, 138, 139, 148, 151, 153]. Screening students at the point of admission may also ensure that those who need supplementary support are identified immediately and referred without delay [34, 37, 73, 74, 79, 92–94, 108, 110, 113, 119, 125, 126, 131, 135, 138, 139, 143, 148, 149, 151, 152]. It was also suggested that offering a range of services and resources throughout the academic trajectory could aid students in overcoming a variety of challenges. These included orientation sessions to the institution and clinical settings, workshops to develop writing, critical analysis and test-taking skills, language courses (specific to nursing), writing/editing support (especially at the graduate level), tutoring services, practical assistance including access to financial aid, scholarships and research funding, social activities, peer support initiatives, a mentorship program and counselling/psychological services [9, 17–20, 33, 34, 37, 50–53, 55, 60, 61, 63–65, 68, 69, 71, 73–77, 79, 80, 82–85, 87–89, 92–105, 107–110, 113–121, 123, 125–133, 138, 139, 141–143, 145–154].

There were also a number of approaches at the curricular/instructor level that were proposed to help students overcome language and cultural barriers and to facilitate learning whether it be in a classroom, clinical or research supervisory context. For example, using audio-visual material, providing information and expectations in writing, giving frequent and detailed feedback, debriefing one on one with students, speaking more slowly and avoiding colloquial language, verifying understanding, using storytelling, audio-taping lectures, and providing more structured guidance (e.g., writing examples for assignments, standardized forms for clinical documentation) [9, 17, 18, 20, 33, 34, 37, 50–52, 60–65, 68, 69, 71, 73–77, 79–85, 87, 88, 90, 92–95, 98–103, 105–108, 110–112, 115, 120, 122–131, 136–143, 145–154]. Evaluations, including assignments and tests could also be modified to accommodate students, for example allowing more time to complete an exam or the opportunity to submit an initial draft of an assignment for feedback before submitting the final version that is to be graded [17, 34, 37, 60, 63, 64, 69, 73, 74, 76, 81, 84, 87, 93, 100, 104, 110, 111, 125, 127, 128, 139, 140, 143, 147, 151, 152]. Course content, evaluations, research topics and clinical experiences may also be adapted to make them more culturally relevant, particularly if students plan to return to work in their country of origin following their graduation [17, 34, 37, 60, 64, 65, 68, 71, 77, 87, 92, 93, 95, 111, 125, 127, 128, 131, 136, 139, 141–143, 145, 147, 148, 152, 154]. Equally emphasized was the importance to provide content and an opportunity to learn more about the host country’s healthcare system and approach to nursing practice [37, 63, 65, 74, 80, 82, 87, 92, 95, 115, 129, 136, 138, 140, 143, 152]. For doctoral students, offering leadership training and opportunities to network and develop their research identity and skills (e.g., conferences, student seminars, research groups, research activities like publishing, committees, joining professional organizations), were also deemed essential [65, 80, 146, 148, 149]. Lastly, to increase foreign-born students’ confidence and feelings of inclusion, it was recommended that instructors foster peer to peer learning and positive interactions between students, show interest in foreign-born students (know their name, relate to them on a personal and emotional level) and be encouraging and respectful [9, 17, 20, 33, 34, 37, 51–53, 55, 65, 69–71, 73–75, 79–84, 87–91, 93–97, 100, 103, 106, 107, 112, 113, 115, 117, 120–125, 127, 128, 130–132, 136, 138–143, 145–154].

### Applying a gender lens

Gender was not explicitly used as a guiding framework or lens, nor was it defined, in any of the studies, literature reviews or discussion papers. Among the research papers that included student participants, 29% did not specify the sex of participants, and although male students were included in 59% of the research (Table 3), overall there were many more female participants compared to male participants across and within studies -over three quarters of the studies with both male and female participants clearly had more females than males. Other gender identities/sexual orientations (e.g., lesbian, gay, non-binary, transgender) were not identified or named in any of the study samples. One study, however, acknowledged that there was a lack of gender diversity among their participants [95].

Of all the studies that included both men and women, only one reported results for the foreign-born students by sex. This study, which examined predictors of success among a cohort of Saudi Arabian students enrolled in an accelerated bachelor program (a collaborative initiative between Saudi Arabia and a US University), showed that the mean graduating grade point average (GPA) varied among female students depending on whether or not they were married or had family present with them in the United States- i.e., single females and women who had no family in the US had lower GPAs compared to their respective counterparts, but these variations were not observed among the male students [66]. One other study and two reviews, which focused on ‘non-traditional students’, also reported results for male nurses, and reported that men tended to feel excluded and delegated to certain roles because of their gender, and felt they were stereotyped as being homosexual [37, 42, 70]. These findings however, did not pertain specifically to foreign-born/ESL/EAL students.

Four quantitative studies included sex as a variable in their analyses with foreign-born students. The study by Carty et al. (2007) showed that overall male students had a higher graduating GPA compared to their female counterparts. A study in Finland with international students found that female students were more likely than male students to perceive cultural diversity in the clinical placement as causing negative consequences, however there were no differences between men and women regarding their perceptions of the impacts of language barriers on their clinical training [109]. Another study, conducted in the US, found sex to not be predictive of attrition among ESL students studying in pre-licensure programs in the state of Texas [72]. Similarly, the fourth study found no association between sex and academic or clinical placement stress among international students studying nursing at the undergraduate level in programs across Australia [100].

With respect to challenges, we identified several papers that reported results and/or that discussed issues related to gender roles and expectations. In one study, conducted more than 30 years ago, a female student participant expressed that it was initially disconcerting, and that it required significant adaptation to come to terms with the idea that women should be assertive and outspoken when interacting with physicians [54]. Similarly, in another study, timidity and not wanting to speak up, was noted to be more challenging for female ESL students compared to male students [84]. In another older study, male students from Saudi Arabia who were studying in the US, found it challenging to have mixed-gender classes, to socialize with female students, to learn about women’s health and to care for female patients in the clinical setting, particularly hygiene and bathing (these same results were also highlighted in a review paper) [17, 64]. Likewise, in another US study (and review), Omani women found it challenging to adapt to openness between sexes, going out alone and independent decision-making [107, 153]. Similar findings were also shown in a study in the UK, where Middle-Eastern women who were completing a doctoral degree, reported finding it difficult to manage everything on their own as they were used to being surrounded by extended family and doing daily activities collectively; consequently these women also reported feeling very lonely [80].

Other difficulties related to gender norms and the mixing of men and women were also reported/discussed, including a hesitation among students to form friendships with the opposite sex because it was deemed inappropriate [81]; male students feeling uncomfortable receiving input or direction from female instructors [84]; female students feeling it is inappropriate to be in ‘intimate’ contact with patients [86]; and women finding it challenging to relate to their native-born female colleagues due to different value systems [97]. The review by Olson (2012) suggested that some female students may not be supported by family during their studies because male family members felt threatened by the possibility that their wives/daughters may earn more income than them [34]. One study also found that female international students more than male students, faced additional challenges professionally post-graduation, irrespective of whether or not they returned to their country of origin, and that these challenges were rooted in the divergent and conflictual cultural norms and expectations of women between the host country and country of origin [84]. Another study supported this notion as it found that international female students from Canada or Europe studying in the US seemed to have less difficulty adjusting to the US compared to other international students due to a greater resemblance in gender norms across the US, Canada and Europe [96].

Managing family/childcare and household responsibilities while studying, and feeling pressure to ascribe to a ‘traditional’ female role, were described as challenges for women in a number of papers [34, 74, 82, 83, 86, 102, 122, 152, 153]. In one study (but highlighted in four different papers), a woman reported significant stress due to her husband and in-laws who disapproved of her studying and who felt that she was a ‘bad wife and mother’ for pursuing her studies [34, 82, 83, 153]. Feeling guilty about leaving children behind also appeared to be a concern particularly affecting women [89, 128]. In contrast, a male student, in the study by Gardner (2005), reported feeling immense pressure to succeed, because he was recognized as a leader in his community in his home country and he felt he needed to return with a nursing degree so that he could help his community [83].

Perceived discrimination was noted in four papers; in one (a research study), an instructor participant reported that a student had shared with her that a patient had said that he did not like the student because the student was ‘a man and foreign’ [74]. In another study, women reported discrimination due to wearing a hijab and being Arab [115]. This latter issue was further highlighted in two review papers [145, 153].

Nursing being perceived as a feminine profession and low status employment was also highlighted as an issue. In one study, a male student participant shared that he felt that his father had concerns about him pursuing nursing as a profession because of his gender [76]. In another study, women from non-English speaking background cultures reported not being supported by their family to pursue their studies in nursing as the profession was deemed to be the type of work that is only done by “loose women or prostitutes” [86].

We did not identify many results or discussion points related to gender and coping. One recent US study, suggests that female students who were mothers found mutual understanding and support from other female students who also had children [76]. Extended family support also seems to be source of help for female students who are trying to balance studying with home/family obligations [34]; in one study a student sent her child to India to be cared for while she completed her studies; providing a better life for her daughter was also a motivating factor that kept her going [83]. Family back home, calling male family members, was also identified as a source of support for the Omani nursing students in the US who were not used to being alone and who found making decisions on their own, challenging [107]. Although not a coping mechanism per se, a number of papers also mentioned that female international students had increased confidence over time and enjoyed the new independence that they had gained while living and studying in the host country [75, 80, 101, 115, 128, 153].

A handful of papers made reference to gender in relation to supportive interventions. One study described a female educator calling on a male colleague to intervene with a male international student on a sensitive topic in order to make the student more comfortable since he was from a cultural background where women usually do not have authoritative roles [84]. Sending letters or involving family members in the orientation was recommended in one study and two reviews, as an approach to enhance family support and understanding for female students who face challenges balancing their studies with family and household responsibilities [34, 83, 152]. Similarly, including fathers and husbands in the admission process was a strategy described in the paper by Robinson et al. (2006) to ensure support for Indian women who wished to pursue their studies in an American university [138]. In the same paper, female applicants were interviewed by women during the recruitment process, and gender dynamics (in reference to male dominance) was considered when pairing female students with community supports once arrived in the US [138]. Matching advisors and international students by sex was also discussed in the paper by Thompson (2012) as an approach to promote comfort for students who are not used to receiving advice from or confiding in someone of the opposite sex [141]. In the study by King et al. (2017), a standardized simulation patient was used as a method to give EAL students an opportunity to get used to providing care to patients of the opposite sex [101]. And avoiding gender bias when presenting exemplars, was given as teaching tip when teaching international students, in the paper by Henderson (2016), [136].

Lastly, gender identity/sexual orientation was not considered or highlighted in any of the results or discussions related to challenges, coping responses or interventions across the literature. The review by Greene et al. (2012) which outlines strategies for promoting the success of international students, however, recommended that students be exposed to and learn how to care for patients with different backgrounds, including different sexual orientations, although no details were provided on how this should be done [33]. The review by Koch et al. (2015) also reported on the clinical placement experiences of lesbian, gay, bisexual, transgender and queer/questioning (LGBTQ) nursing students, but this was for nursing students in general and not specific to foreign-born/ESL/EAL students [42]. The review highlighted that overall there is very little known about the experiences of LGBTQ nursing students.

## Discussion

Overall, the literature mostly reflects women’s experiences, there was less focus on men, and students who identify as other genders/sexual orientations were not visible in the research and discussions. Our review shows that international and migrant nursing students face a number of challenges associated with different cultural roles, norms and expectations for men and women; other challenges included perceived discrimination, and in general nursing being viewed as a feminine, low-status profession. We only identified a couple of strategies, accessing support from family and other student mothers, used specifically by female students to cope with some of the challenges associated with gender roles and norms, and we found nothing regarding men’s coping responses. Supportive interventions that considered gender were limited; these included matching students with support persons and advisors by sex, involving male family members in admission and orientation processes, and using patient simulation as a method to prepare students for care-provision of patients of the opposite-sex. Taken together, the results reveal that sex, gender and gender identity/sexual orientation have been under examined and discussed in the literature on international and migrant nursing students in academic institutions in major host countries.

Equity, diversity and inclusion (EDI) are fundamental to the nursing profession and its practice as nurses interface with individuals, families and communities in very intimate ways (physically, psychologically, socially and spiritually/existentially) and during the most vulnerable moments of life, which are greatly influenced by one’s social group memberships/identities such as gender, culture, religion, ethnicity and sexuality. Therefore in order to promote the health and well-being for all, nurses must be prepared to respond to the needs of diverse populations and to provide care that is safe and that addresses inequities. It also requires a workforce that reflects the population demographics. Despite there being a movement towards inclusivity, the profession, however, remains predominantly Caucasian (in high-income countries) and heteronormative, especially at the leadership levels, and gender and gender identity/sexual orientation discrimination are still prevalent [39, 155–158]. Rectifying this problem begins with nursing education programs that are inclusive, fair, and that celebrate diversity within the curriculum, and among the student, faculty and administrative bodies. EDI are currently top priority for many academic institutions in major migrant/international student receiving-countries [159–161]. Many have developed strategic plans and have a mandate to implement strategies to reduce discrimination and bias and create more respectful learning environments where the presence and expression of differences are valued and supported and everyone feels they belong and can thrive. Gender issues in higher education and the need for gender-sensitive interventions at the structural and curricular levels in order to attract and retain students, have been identified in both the nursing and international education literature, respectively [25, 30, 31, 36, 162–164]; more recently, there has also been greater attention given to gender identity/sexual orientation [40, 41, 43, 45, 165]. To further develop EDI best practices, and to advance the profession and practice, future research and discussion papers in nursing higher education must also address the intersections of gender, gender identity/sexual orientation and foreign-born status.

The review also highlights that a variety of terms have been used in the literature to describe foreign-born students, and that although migrant students have been included to some extent, the role of migration history/status and length of time in country have not been considered, making it difficult to tease out information about groups in more vulnerable contexts. More recently-arrived migrants are more likely compared to more established migrants to experience cultural barriers, be unfamiliar with a host country’s systems and have difficulty accessing services. Many refugees and asylum-seekers have experienced trauma and difficult migration trajectories that can exacerbate mental health issues and further complicate adjustment to a new academic environment [166–168]. Refugees and asylum-seekers are also more likely compared to other migrants and international students to have experienced disruptions in their education and to face language barriers and social-economic disadvantages during resettlement [166–168]. They are also more likely to experience family separation and may feel greater pressure to succeed especially if family members in the home country are financially dependent on them. Asylum-seekers also are commonly excluded from social programs and have the added strain of not knowing what their future holds. Therefore to have a more nuanced understanding of foreign-born nursing students’ challenges and coping responses, and to better identify supportive interventions, future work should take into account not only gender and gender identity/sexual orientation, but also the migration context (status and length of time), which should be clearly defined [41, 169–172].

The results of the review show that generally there is an abundance written on supportive interventions for foreign-born nursing students in academic institutions, however it remains mostly descriptive and anecdotal. The results raise a number of questions regarding the specifics on how institutions and educators can best be supportive. For example, language and communication remain significant issues yet it is unclear what level of language ability should be required upon admission- high level requirements restrict access while a low level requirement puts undue stress on students, particularly since nursing requires knowledge of specific terminology. Likewise, to what extent should educators adapt teaching approaches and evaluations to facilitate adjustment to the new academic milieu and how can educators effectively provide emotional support whilst maintaining their professional stance? Furthermore, what should the role of institutions be in ensuring that foreign-born students are adequately prepared for work post-graduation, whether they stay in the host country or decide to return to their country of origin, especially when increasingly these institutions are integrating notions of EDI in their mandates? For example, should institutions provide additional support to prepare foreign-born students for the licensure exam in the host country? Alternatively, should institutions provide training within the program that is relevant to international students’ country of origin context and/or provide re-entry programs prior to students’ return home? It would be timely to also study and debate these broader questions related to supportive interventions.

### Limitations and strengths

We purposely chose to not use ‘gender’, ‘gender identity’ or related terms in our search strategy so that our search would be broad, however, this exclusion may have contributed to missing some literature. We did not include grey literature, which may explain the lack of language diversity (French and Spanish publications), and consequently the small number of papers on students’ experiences in non-English speaking host countries. Due to the scoping nature of the review we did not closely analyze or report on the evidence related to the identified challenges, coping responses and supportive interventions (e.g., prevalence of challenges, evaluations of interventions). We also did not report on the overall benefits or positive experiences of foreign-born nursing students, which would have been informative. Nevertheless, the review was very thorough and provides a comprehensive overview with a gender lens, of the challenges, coping strategies and supportive interventions that have been studied and discussed over a 39 year period. The results also highlight gaps in the literature, especially with regards to gender.

### Future research

Future research on challenges, coping responses and supportive interventions for international and migrant students in academic nursing programs in major host countries, should include sex and gender-based analyses; an intersectionality-based approach, including gender, gender identity/sexual orientation, migration/international status and context, as well as other identity markers (e.g., race, religion) is warranted. Additional reviews on existing gender and gender-identity/sexual orientation sensitive interventions in nursing/healthcare education in general, and for foreign-born students across a variety of disciplines, would also be informative. Overall, more studies that test and evaluate supportive interventions for international and migrant nursing students, at both the structural and curricular levels are needed; a systematic review would be useful as well to provide a better evidence base for academic institutions to draw from. Since most of the literature to date has focused on the US context, and much more has been written on undergraduate students, more research in non-English speaking countries, and with graduate students, especially at the doctoral level, would also be worthwhile.

## Conclusion

The literature on the challenges, coping responses and supportive interventions for international and migrant students in academic nursing programs in major host countries, has significant gaps with regards to how it addresses the contributions and consequences of sex, gender and gender identity/sexual orientation related experiences. To draw and retain a diversity of candidates to the nursing profession, and to create more inclusive and equitable learning environments, future work, especially with respect to supportive interventions, needs to address the intersections of gender, gender identity/sexual orientation and foreign-born status, and also consider the complexity of migrant students’ contexts.

## Supplementary Information

**Additional file 1.** Database search results.

## Data Availability

All data generated and analysed during this study are included in this published article and the original sources.

## Abbreviations

CALD: Culturally and linguistically diverse
CINAHL: Cumulative Index to Nursing & Allied Health Literature
EAL: English as an additional language
EBP: Evidence-based practice
EDI: Equity, diversity and inclusion
Embase: Excerpta Medica dataBASE
ERIC: Education Resources Information Centre
ESL: English as a second language
GPA: Grade point average
LGBTQ: Lesbian, gay, bisexual, transgender and queer/questioning
UK: United Kingdom
US: United States
