# Supplementary material for: Challenges, coping responses and supportive interventions for international and migrant students in academic nursing programs in major host countries: a scoping review with a gender lens
Source: BMC Nurs. 2021 Sep 18;20:174. doi: 10.1186/s12912-021-00678-0 (PMC8449499; doi:10.1186/s12912-021-00678-0)
Supplement: Supplementary file 1 — Additional file 1. Database search results. [file 12912_2021_678_MOESM1_ESM.docx]

**Additional File 1: Database search results**

EMBASE: 1547

MEDLINE: 1268
PsycINFO: 699
CINAHL: 2074
Cochrane: 22
ProQuest: 347
Sociological abstracts: 40
Web of Science: 727

Joanna-Briggs: 39

Eric: 1506
